# Assessing overdiagnosis of fecal immunological test screening for colorectal cancer with a digital twin approach

**DOI:** 10.1038/s41746-023-00763-5

**Published:** 2023-02-10

**Authors:** Ting-Yu Lin, Sherry Yueh-Hsia Chiu, Ling-Chun Liao, Sam Li-Sheng Chen, Han-Mo Chiu, Tony Hsiu-Hsi Chen

**Affiliations:** 1 Institute of Epidemiology and Preventive Medicine, College of Public Health, National Taiwan University, Taipei, Taiwan; 2 Department of Health Care Management, College of Management, Chang Gung University, Taoyuan, Taiwan; 3 Division of Hepato-Gastroenterology, Department of Internal Medicine, Kaohsiung Chang Gung Memorial Hospital, Kaohsiung, Taiwan; 4 Department of Pharmacy, National Taiwan University Hsin-Chu Hospital, Hsinchu, Taiwan; 5 School of Oral Hygiene, College of Oral Medicine, Taipei Medical University, Taipei, Taiwan; 6 Departments of Internal Medicine, National Taiwan University Hospital, Taipei, Taiwan

**Keywords:** Epidemiology, Colon cancer

## Abstract

Evaluating the magnitude of overdiagnosis associated with stool-based service screening for colorectal cancer (CRC) beyond a randomized controlled trial is often intractable and understudied. We aim to estimate the proportion of overdiagnosis in population-based service screening programs for CRC with the fecal immunochemical test (FIT). The natural process of overdiagnosis-embedded disease was first built up to learn transition parameters that quantify the pathway of non-progressive and progressive screen-detected cases calibrated with sensitivity, while also taking competing mortality into account. The Markov algorithms were then developed for estimating these transition parameters based on Taiwan FIT service CRC screening data on 5,417,699 residents aged 50–69 years from 2004 to 2014. Following the digital twin design with the parallel universe structure for emulating the randomized controlled trial, the screened twin, mirroring the control group without screening, was virtually recreated by the application of the above-mentioned trained parameters to predict CRC cases containing overdiagnosis. The ratio of the predicted CRCs derived from the screened twin to the observed CRCs of the control group minus 1 was imputed to measure the extent of overdiagnosis. The extent of overdiagnosis for invasive CRCs resulting from FIT screening is 4.16% (95% CI: 2.61–5.78%). The corresponding figure is increased to 9.90% (95% CI: 8.41–11.42%) for including high grade dysplasia (HGD) and further inflated to 15.83% (95% CI: 15.23–16.46%) when the removal adenoma is considered. The modest proportion of overdiagnosis modelled by the digital twin method, dispensing with the randomized controlled trial design, suggests the harm done to population-based FIT service screening is negligible.

## Introduction

The efficacy of the guaiac fecal occult blood test (gFOBT) for colorectal cancer (CRC) screening has been proven by four randomized controlled trials (RCT) on gFOBT^1–4^ and three service screening programs on the fecal immunological test (FIT)^5–7^ but there still exists large uncertainty of overdiagnosis^8^, which is one of the demerits of population-based service screening that has already been noted in the PSA (prostate-specific antigen)^9–11^.

**Fig. 1:**
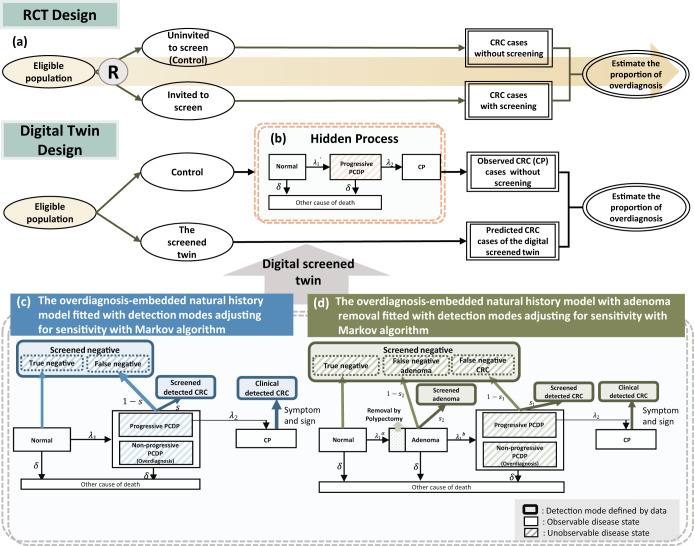
Randomized controlled trial (RCT) and digital twin design for overdiagnosis in population-based screening. **a** The design of randomized controlled trial for evaluation of overdiagnosis; **b** the hidden disease natural history process from normal, PCDP, until the clinical phase among the control group in the absence of screening; **c** the overdiagnosis-embedded multi-state Markov model and algorithms for learning parameters with adjustment for sensitivity and competing mortality from the screened group; **d** the overdiagnosis-embedded multi-state Markov model and algorithms with adenoma for learning parameters with adjustment for sensitivity and competing mortality from the screened group.

Previous studies on population-based CRC screening with gFOBT found 2%-13% overdiagnosis when the incidence method was applied to data from both the Nottingham trial and the Finnish trial^12,13^. Such an incidence method is valuable but must rely on the use of randomized controlled trial (RCT) data to compare the expected CRC cases derived from the screened group with those from the control group as shown in Fig. 1a. While g-FOBT has data on RCT, it still lacks RCT data for FIT screening. Evaluating overdiagnosis in two-stage FIT service screening for CRC is therefore faced with these obstacles.

The alternative to the RCT approach for assessing the extent of overdiagnosis in service screening program is to use the modelling approach as already applied to PSA screening for prostate cancer^10,14,15^, which takes into account the complex natural course of tumor development that is inherent to a multi-stage biological process due to environmental exposures and genetic susceptibility^16^, which may either accelerate or slow down the process, making allowance for competing mortality. Therefore, the definition of overdiagnosis not only comprises non-progressive cancers that develop slowly and remain in the pre-clinical state (extremely slowly-growing tumor) but also progressive cancers that were detected in the pre-clinical state but died from other causes^17^. In addition to using a computer simulation approach, quantifying the extent of overdiagnosis can be escalated up to a study design level in conjunction with machine learning algorithms for estimating relevant parameters governing the overdiagnosis-embedded disease natural process. One approach relies on the recently proposed digital twin approach, which arose from the quantified-self phenomenon to promote the value of self-monitoring made possible by emerging digital technologies for data acquisition and self-reflection^18^. Its applications include international space station^19^, biology^20^, and medicine^21^. More importantly, the concept of the digital twin was realized in the realm of cancer prevention and screening by the parallel universe approach, which has already been used in a micro-simulation scenario for the development of CISNET (Cancer Intervention and Surveillance Modeling Network)^22^. The parallel universe structure is first to simulate the population in the absence of the screening and then simulate the identical population with the uptake of screening in order to evaluate the effectiveness of screening program^23,24^. The parallel universe approach, as shown in Fig. 1a, can be used to evaluate overdiagnosis by first mirroring real-world CRC cases arising from the underlying eligible population in a no-screening world and then virtually recreating the digital twin with the identical population subjected to screening. However, to build up such a digital twin requires the development of computer algorithms for learning parameters that govern the unobservable overdiagnosis-embedded disease natural history process.

Doing so can tackle the thorny issue of the unobservable disease natural process of distinguishing non-progressive cancers from progressive cancers that stay in the pre-clinical detectable phase (PCDP) due to the interruption of treatment. Moreover, in stool-based two-tier screening modelling for population-based service screening data on overdiagnosis through the disease natural history process would be affected by the sensitivity of the gFOBT or FIT^25^. The failure to calibrate the sensitivity of a test may lead to a biased estimation of overdiagnosis. Moreover, one also needs to consider the competing causes of death, which is another cause of overdiagnosis for both non-progressive and progressive cancers.

The aim of this study is therefore to first build up the overdiagnosis-embedded disease natural process governed by transition parameters and the calibrated sensitivity, while accounting for competing mortality. The corresponding Markov algorithms were developed to estimate these parameters based on the data of the population-based organized service program in Taiwan. The digital screened twin, mirroring the control group, was virtually created to predict CRC cases with the trained parameters in relation to the overdiagnosis-embedded natural process. Those predicted cases were further compared with the real-world data on the observed CRCs for assessing the extent of overdiagnosis of CRC related to FIT screening. To have external validation of this approach, we also applied this digital twin method to gFOBT with the available data from two RCTs in the UK and Denmark, respectively.

The findings show the extent of overdiagnosis for invasive CRCs resulting from FIT screening drops from 9.90% (95% CI: 8.41–11.42%) to 4.16% (95% CI: 2.61–5.78%) with and without allowing for high-grade dysplasia (HGD), respectively. The corresponding figure is further inflated to 15.83% (95% CI: 15.23–16.46%), taking the removal of adenoma into account. With such a low proportion of overdiagnosis predicted by the digital twin method, it appears that population-based FIT service screening has suffered minimal harm.

## Results

### Markov model learning process calibrated by sensitivity

Before the digital twin of 5,417,699 subjects eligible for FIT service screening in Taiwan can be virtually recreated to impute the expected CRC cases for estimating the proportion of overdiagnosis, it is necessary to learn relevant parameters from the overdiagnosis-embedded disease natural history estimated by the Markov-based algorithm. We first applied the multi-state Markov model and algorithms for learning two transition parameters from this screened group shown in Fig. 1c, yielding 1.41 (per 1000 person-years) of the pre-clinical rate and 0.39 (95% CI: 0.37, 0.40) of the annual progression rate from PCDP to CP, equal to 2.59 (95% CI: 2.50, 2.68) of mean sojourn time (MST). The sensitivity was estimated as 80.25% (95% CI: 78.12%-82.32%). The lower panel of Table 1 shows the results of the corresponding estimates using the overdiagnosis-embedded disease natural history while making allowance for the influence of the removal of adenoma.

**Table 1:** Estimated results of overdiagnosis-embedded disease natural history model without and with consideration of adenoma removal in three studies.

Parameters	λ_1_ (Normal → PCDP)	λ_2_ (PCDP → Clinical)	Sensitivity^c^	Mean sojourn time (1/λ_2_)	
Without consideration of Adenoma removal					
Taiwan population-based study^a^	0.00141 (0.00139, 0.00143)	0.3860 (0.3726, 0.3997)	80.25% (78.12%, 82.32%)	2.59 (2.50, 2.68)	
UK trial	0.00147 (0.00136, 0.00159)	0.3475 (0.2437, 0.4513)	53.40% (34.26%, 69.55%)	2.88 (2.22, 4.10)	
Demark trial	0.00172 (0.00155, 0.00189)	0.4433 (0.3226, 0.5639)	52.05% (35.53%, 68.56%)	2.26 (1.77, 3.10)	
	λ_1_^a^ (Normal → Adenoma)	λ_1_^b^(Adenoma → PCDP)	λ_2_(PCDP → CRC)	Sensitivity^d^	Sensitivity^c^
With consideration of Adenoma removal					
Taiwan population-based study^b^	0.00149 (0.00149, 0.00150)	0.0839 (0.0831, 0.0846)	0.5300 (0.5223, 0.5381)	63.48% (59.89%, 67.05%)	82.86% (78.20%, 88.77%)

^a^Without consideration of adenoma.

^b^With consideration of adenoma.

^c^Sensitivity of PCDP CRC detection.

^d^Sensitivity of Adenoma detection.

### Estimating the proportion of overdiagnosis of CRC screening

To estimate the proportion of overdiagnosis, we applied Equation (14) and Equation (15) in Supplementary Methods with the application of parameters to impute the CRC cases derived from the screened twin in comparison with the unscreened twin. For FIT-based Taiwan population-based screening, the expected CRCs were 79,469 and 84,425 without and with considering adenoma removed by polypectomy, as shown in as shown in Fig. 1c and d, which are larger than the adjusted observed 72,587 CRC cases, making allowance for the increasing incidence rate projected from the pre-screening period. When only invasive cancer was considered, the proportion of overdiagnosis as a result of FIT-based population-based screening in Taiwan was 4.16% (95% CI: 2.61–5.78%), but this increased to 9.90% (95% CI: 8.41%-11.42%) when high-grade dysplasia (HGD) was included. The overdiagnosis after further consideration of the removal of the adenoma was inflated to 15.83% (95% CI: 15.23–16.46%) (Table 2).

**Table 2:** Estimated the proportion of overdiagnosis based on overdiagnosis-embedded disease natural history model in three studies.

Study	Control group population	Mean of Follow-up time	Predicted CRC from the digital screened twin, E(t)	Observed CRC from the control group, U(t)	Overdiagnosis (%) (95% CI)
Invasive cancer only					
Taiwan population-based study^a^	5,417,699	11.0	66,339	63,616	4.16 (2.61,5.78)^c^
Including HGD					
Taiwan population-based study^a^	5,417,699	11.0	79,469	72,587	9.90 (8.41,11.42)^c^
UK trial	74,998	8.5	931	856	8.79 (8.28,9.65)
Demark trial	30,966	10.0	528	483	9.33 (8.81,10.20)
With consideration of adenoma removal					
Taiwan population-based study^b^	5,417,699	11.0	84,425	72,587	15.83 (15.23, 16.46)^c^

^a^Without consideration of adenoma.

^b^With consideration of adenoma.

^c^With consideration of competing risk of death.

### Applications to two randomized controlled trials on overdiagnosis of gFOBT

Two RCT data as shown in Table 3 were used for learning parameters in relation to the imputation of the predicted cases, including progressive cancers and non-progressive cancers, as a result of the screen, as shown in Fig. 1. The upper panel of Table 2 shows that the preclinical incidence rates of RCTs with gFOBT were 1.47 and 1.72 (per 1000 person-years) for the UK and Denmark trials, respectively, and the corresponding figures of the estimated MST were 2.88 (95% CI = 2.22–4.10) and 2.26 (95% CI = 1.77–3.10) years. In addition, the estimated sensitivities of gFOBT were 53.40% (95% CI = 34.26–69.55%) for the UK trial and 52.05% (95% CI = 35.53–68.56%) for the Denmark trial.

**Table 3:** Number of CRC by detection modes and model validity with the overdiagnosis-embedded disease natural history model of two gFOBT randomized controlled trials of CRC screening.

Parameter	Status	Nottingham trial (UK)		Funen trial (Demark)	
Screening finding by round		Observed	Predicted	Observed	Predicted
Prevalent screening	Normal	44733	44735.42	20635	20630.24
	CRC	104	101.58	37	41.76
Interval cancer	CRC	164	140.04	148	147.23
Positive but without confirmation (first round)	CRC	28	27.76	----	----
Positive but without confirmation (repeated round)	CRC	57	66.03	----	----
Repeated screening	Normal	88008	87977.51	66025	66014.12
	CRC	132	109.69	83	67.74
Refuser	Normal	30015	30014.06	9895	9895.71
	CRC	400	400.94	195	194.29
Internal validity Goodness of fit: Chi-square		9.9421 (d.f.=6)		3.9895 (d.f.=4)	
		(*p* value = 0.1271)		(*p* value = 0.4074)	

For checking the adequacy of the proposed Markov learning model, the results of goodness of fit show that there was a lack of statistical significance with respect to the comparison between the expected and observed number of CRCs by each detection mode in two RCTs (all p-values > 0.05; Table 3). These findings suggest that the Markov model and algorithm for learning the overdiagnosis-embedded disease natural history with adjustments for sensitivity and competing mortality may be adequate to estimate the proportion of overdiagnosis.

Table 2 also shows the corresponding results of the expected CRCs in comparison with the observed CRCs. In two RCTs, the proportions of overdiagnosis due to gFOBT using the control group were 8.79% (95% CI: 8.29%-9.65%) and 9.33% (8.81%-10.20%), respectively, for the UK and Denmark trials, which are between the 2% and 13% reported in previous studies using the incidence method^12,13^.

## Discussion

In contrast to 20% to 60% overdiagnosis^26–28^ observed in PSA screening for prostate cancer, the proportion of overdiagnosis for FIT population-based screening is modest, implying that the harm done to the population-based FIT service screening program is as minor seen with gFOBT. Furthermore, when only invasive cancers were included, the estimated proportion of overdiagnosis based on FIT population-based screening decreased from 9.90% to 4.16%. This finding implies that more than half of overdiagnosed CRCs were derived from HGD.

The other factor that might affect the result of overdiagnosis is the sensitivity of the test (gFOBT/FIT) which is inversely associated with the MST. Our modelling approach took sensitivity into account, and the results of the estimated sensitivities were 53.40% and 52.05% in the UK and Denmark trials, respectively. According to the previous report of the UK trial, the estimated sensitivity of gFOBT^2^ was 53.4% by using the proportional incidence method, which was very close to our results. Our application of the overdiagnosis-embedded disease natural history model of CRC calibrated with sensitivity would render the proportion of overdiagnosis more accurate as sensitivity is also affected by the length of MST. It should be noted that the long MST is often associated with overdiagnosis if the length of follow-up time is neglected. Since sensitivity and overdiagnosis cannot be observed directly, using the overdiagnosis-embedded disease natural history model, making allowance for sensitivity, can deal with this issue to estimate both parameters in conjunction with the pre-clinical incidence rate.

The underlying assumption of this model is that the overdiagnosed cases would not progress to CP, as shown in Fig. 1c and d mainly, including nonprogressive cancers but also progressive cancers dying from other causes of death. The sojourn time by definition is therefore infinite after the lesion entered PCDP. However, these over-detected cases derived from the control group would not be observed in the absence of screening. That accounts for why we can apply this model to estimate the proportion of overdiagnosis.

Moreover, one important characteristic of CRC screening is that the early detection of adenoma followed by its removal through polypectomy might reduce the incidence of CRC. Without considering adenoma removal, the risk of overdiagnosis may be underestimated. The advantage of using the overdiagnosis-embedded disease natural history with the incorporation of adenoma is to offset overdiagnosis contributed by the removal of adenoma by polypectomy in two-tier stool-based screening to re-estimate the incidence rate and the inflated proportion of overdiagnosis. It should be noted that the program sensitivity for adenoma may vary with different population-based screening programs^29^, particularly depending on the ability to detect small or advanced adenoma by colonoscopy, which would also be reflected in subsequent screens for catching up on those missed adenomas. To assess how the calibration of sensitivity on adenoma will offset the adenoma removed by polypectomy, we did a series of sensitivity analyses by changing the parameters of sensitivity from 20% to 63%. The results show a small change from 13% to 16%. This means the consideration of adenoma removed by polypectomy with different estimates of sensitivity for detecting adenoma would only lead to a slight change in our estimate of overdiagnosis.

From the aspect of the methodology, although our developed Markov algorithm used in digital twin design as shown in Fig. 1 enables one to estimate the extent of overdiagnosis of cancer screening here it is not only the sole approach. In analogue to the digital twin approach, the parallel universe approach previously proposed for the development of CISNET is also a very useful means for evaluating the extent of overdiagnosis and would do the same task if it is applied to our data on FIT service screening^23,24^. The comparison of the results on different machine learning algorithms for evaluating effectiveness and overdiagnosis would enhance the verification of the digital twin design and the parallel universe structure alternative to the traditional RCT design. This would become one of the ongoing research projects in the realm of digital health.

There are several limitations to this study. First, although we consider the influence of adenoma removed by polypectomy, this does not mean we estimated the proportion of overdiagnosed adenoma, as two-tier stool-based screening can only have early detection of a proportion of adenoma rather than the whole group of adenoma. To estimate the true proportion of overdiagnosis of adenoma, it requires the same overdiagnosis-embedded model to estimate the transition parameters based on the screened group data from primary screening with colonoscopy. Otherwise, it requires long-term follow-up to remove nuisance factors such as the awareness of detecting adenoma inroutine clinical practice that may affect the assessment of overdiagnosis. The second limitation is that estimating the proportion of overdiagnosis is dependent on control group information. However, in the population-based organized service screening program, there is a lack of control group without screening. Alternatively, we used the data from the screened group to project the number of cancers in comparison with the observed number using data from the pre-screening period with an adjustment for the increasing incidence rate. Whether the comparator from the pre-screening period is valid should be validated by other data on population-based service screening programs like two well-reputed ones, the Italian or Kaiser Permanente programs, both of which have demonstrated the effectiveness of the FIT screening^5,6^. The third limitation is that the cut-off of FIT test would determine the sensitivity, which further affects the extent of detecting non-progressive cancers (Fig. 1c and d) and the proportion of overdiagnosis. In Taiwan, the government set the threshold of 20 μg per gram of stool as the cutoff for the FIT screening program. The estimated parameters may not be directly applied to other programs with different the cut-offs. However, the proposed digital twin is still applicable if the change of sensitivity parameters in response to the alteration of cut-off can be adjusted in the overdiagnosis-embedded natural history process. The fourth concern is the psychosocial impact of overdiagnosis. Even though the proportion of overdiagnosis of CRC was estimated to be lower than 10%, there are still some concerns about anxiety and unnecessary treatment and surveillance which might cause additional economic cost and increase the risk of side effects and complications such as perforation^30^. However, the scope is beyond the context of this paper.

In conclusion, overdiagnosis of CRC with FIT in population-based service screening evaluated by the digital twin approach is negligible. This finding implies the FIT test is less likely to lead to unnecessary colonoscopies and treatments when it is offered in a population-based CRC service screening program.

## Methods

### Digital twin design

We evaluated the phenotype of overdiagnosis resulting from stool-based mass screening, with the study design borrowing from the concept of the digital twin as mentioned above. Before proposing the digital twin design, it is necessary to introduce the traditional RCT design as shown in Fig. 1a for analysis of overdiagnosis. The RCT is the standard design for evaluating whether FIT screening is effective in reducing mortality from CRC and whether there is overdiagnosis resulting from FIT screening, but it may not throw light on how overdiagnosis is invoked because the RCT neither elucidates the hidden disease natural history process from normal, through the occult pre-clinical detectable phase (PCDP) until the clinical phase in the control group in the absence of screening (Fig. 1b). Nor can the pathways leading to overdiagnosis in the screened group (Fig. 1c and d) be elucidated. Therefore, even though the result of overdiagnosis can be achieved with the RCT, the overdiagnosed cancers are often mixed up with progressive cancers detected in the pre-clinical detectable phase (PCDP) if the follow-up time is not long enough to wash out these early-detected progressive cases because of the lead time that would advance the date of diagnosis. Moreover, sensitivity is the other factor affecting the estimate of overdiagnosis when it comes to population-based service screening rather than the RCT program because the sensitivity of FIT would affect the estimate of overdiagnosis, but such an influence can’t be directly observed.

To assess the extent of overdiagnosis in population-based service screening, we need to consider the separation of lead-time gained progressive cancers from non-progressive cancers and the sensitivity of FIT. The first initiative is to build up the overdiagnosis-embedded disease natural history model of CRC to distinguish progressive cancers from non-progressive ones staying in pre-clinical detectable phase (PCDP) (Fig. 1c) and then to model the unobservable process from PCDP to clinical phase (CP) for progressive cancers due to the interruption of treatment and also the removal of adenoma by polypectomy (Fig. 1d). Allowance would also be made for the sensitivity of FIT in Fig. 1c and d in order to fit different detection modes defined by the data. Relevant transition parameters include the incidence rate of PCDP (including progressive and non-progressive phenotypes), the progression rate from progressive PCDP to CP without or with considering the removal of adenoma by polypectomy, as shown in Fig. 1c and d, and sensitivity. These parameters were then trained by fitting the developed Markov machining algorithm to population-based FIT service screening data with different detection modes, as information on overdiagnosis can only be learned from those with the uptake of screening for detecting both progressive and non-progressive CRC in the PCDP as shown in Fig. 1c and d. Note that Markov algorithms developed for learning these transition parameters are detailed in the statistical section.

The spirit of the digital twin design is to emulate the RCT design to yield CRCs in the invited group and the control group through the pathways of multistate disease’s natural process, as opposed to the conventional RCT design without the detailed underlying disease process. To create the mirror of the control group without screening, these trained transition parameters were further applied to generating a virtually-created digital screened twin for predicting CRCs in the identical control group subjected to screening (Fig. 1c and d). The observed CRC cases, if there are symptoms and signs related to CRC, from the control group would end up being diagnosed as CRC in the CP following the hidden natural course of Fig. 1b from normal through the occult PCDP until the CP. Without screening, PCDP cannot be detected, nor can overdiagnosed CRC be detected in the control group. The comparison was therefore made between the predicted CRCs of the digitally screened twin and the observed CRCs of the control group in the absence of screening.

### The underlying mechanism for quantifying the proportion of overdiagnosis

The incidence rate of PCDP in Fig. 1c (denoted by λ_1_) is thought to be higher than in Fig. 1b (denoted by λ_1_') due to an excess of nonprogressive PCDP cancers detected by screen. Cancers predicted on the basis of the virtual, digitally screened twin after applying the transition parameters learned from Fig. 1c are therefore expected to be larger than their counterparts in the control group following the natural history of Fig. 1b in the absence of screening. The excess of proportion, as calculated in the statistical section, reflects the extent of overdiagnosis.

Overdiagnosis would be underestimated if the removal of adenoma was not considered because screening not only interrupts the natural history from PCDP to CP but also stops the malignant transformation from adenoma to cancer via polypectomy. In order to adjust for such an influence, Fig. 1d shows how to apply the multi-state model, namely Normal→ Adenoma→ PCDP→ CP, to estimating three parameters in order to offset overdiagnosis contributed by the removal of adenoma by polypectomy. The predicted of CRCs of the digital screened twin based on three transition parameters (λ_1_^a^,λ_1_^b^, and λ_2)_ in Fig. 1d were compared with the observed CRCs of the control group in the absence of screening following the hidden natural history of Fig. 1b to calculate the proportion of overdiagnosis adjusting for the effect of removal of adenoma. Note that competing mortality, one of the causes related to overdiagnosis, was also considered for both progressive and non-progressive stays in the PCDP.

### Data source

#### Taiwan Population-based CRC Screening Program with Fecal Immunochemical Test (FIT)

Population-based CRC screening data were collected from the Taiwan Colorectal Cancer Screening Program, which was launched by the Taiwanese government in 2004 and provided a biennial FIT screening for residents aged 50–69 (the upper age limit was extended to 74 after 2013). Positive cases were referred for diagnostic colonoscopy if the hemoglobin concentration was greater than 20 μg per gram of stool (equivalent to 100 ng/mL for the Eiken OC-Sensor and 8 ng/mL for the Kyowa HM-JACK)^31^. For those subjects with negative result, they were advised to participate in the subsequent round of screening two years later. All screening data and the examination findings including adenoma were collected in the centralized database of the organized service screening program under the auspices of the health authority. Information on interval CRCs and CRCs from FIT screening non-participants were obtained from the National Death Registry and the Taiwan Cancer Registry according to International Classification of Diseases (ICD), 9th revision. The population cohort recruited 5,417,699 eligible population aged 50 to 69 years during the period of 2004–2009 and there were 3,811,011 participants in this program. The cohort was followed up on until 2014 to determine cancer status via a link to Taiwan’s national cancer registry. A total of 71,543 CRCs were identified, including 64,199 invasive CRCs and 7,344 HGD, which were used for assessing the proportion of overdiagnosis for invasive CRCs only and all CRCs. By detection modes, there were 13,821 screen-detected CRCs, 11,904 interval cancers, and 45,818 CRCs among non-participants. The cohort was divided into two scenarios based on the overdiagnosis-embedded natural history process without considering the removal of adenoma following Fig. 1c and the overdiagnosis-embedded natural history process with the consideration of the removal of adenoma following Fig. 1d. Because population-based service screening does not have a control arm as designed in the RCT, we used the pre-screen period data (approximate 5,417,699 population size) between 1998 and 2003 before nationwide service screening as the comparator, with an adjustment for the increasing incidence trend of CRC that would capture the biological growth rate of CRC incidence (4.5% per year) in the absence of screening^7^. This increasing trend was further applied to imputing the observed CRCs of this control group without screening in parallel with the contemporaneous period of the screened group serving between 2004 and 2014, as mentioned above. This study was approved by the Health Promotion Administration of the Ministry of Health and Welfare of the Taiwanese government, and informed consent was waived for the deidentified data.

### Statistical analysis

We proposed a Markov process to model the overdiagnosis-embedded natural history process in Figures c and d. Here, we learned the corresponding parameters based on population-based FIT screening data in Taiwan that consisted of both progressive and non-progressive PCDP cancers. Because the screening test (i.g. gFOBT or FIT) may miss the lesion, we took the sensitivity into account to adjust the annual progression rate (λ_2_)^32,33^. As the competing risk for other causes of death (δ) is also one of the causes accounting for overdiagnosis, this state was incorporated into the overdiagnosis-embedded natural model in Figures c and d. Supplementary Methods give the details of how to estimate the relevant parameters with the Markov-based algorithms, namely the likelihood functions, of the multistate model after learning from the empirical data on population-based service screening by detection modes^34^. The Bayesian MCMC algorithm with inverse-gamma (0.001, 0.001) prior distributions for the transition parameters is used for estimating the parameters for multi-state Markov model, and the 95% credible interval is further obtained from the posterior distribution of each parameter.

As mentioned earlier, due to the overdiagnosis of CRCs, it is postulated that the annual incidence rate of CRC (λ_1_) applied to the digitally screened twin would be greater than that (λ_1_^'^) following the hidden natural history process arising from the control group in the absence of screening. CRC cases derived from the screened twin (S) were virtually imputed by the already learned transition probabilities of different types of detection modes, as mentioned above, with the identical follow-up time of the control group. Therefore, we then imputed the ratio of the predicted CRCs of the screened twin (S(t)) to the observed (U(t)) CRCs of the control group during time t, which can be expressed by S(t)/U(t). Then, the proportion of overdiagnosis of CRC given time t can be calculated as (S(t)/U(t)-1)×100%, representing the extra proportion of screening-detected cases that would not have been diagnosed in the absence of screening. Furthermore, with the influence of the adenoma removed by polypectomy in two-tier stool-based screening, the predicted CRCs (S) are recalculated by applying the transition parameters as shown in Fig. 1d. The detailed procedure for quantifying the proportion of overdiagnosis refers to Supplementary Methods.

We used two gFOBT randomized controlled trials to examine whether the predicted numbers of CRC based on the proposed Markov algorithm are in good agreement with the observed numbers of CRC by the detection modes using the goodness of fit of Pearson Chi-square test to validate whether the Markov-based algorithms for virtually re-creating the digital twin are adequate. The estimated proportion of overdiagnosis using the proposed digital twin method was also calculated for the gFOBT test in comparison with those estimates based on the randomized controlled trial data. The details of aggregate data by detection mode are listed in Table 3. An illustration of calculating the proportion of overdiagnosis using the UK trial on gFOBT is also specified in Supplementary Methods.

### Reporting summary

Further information on research design is available in the Nature Research Reporting Summary linked to this article.

## Supplementary information

Reporting Summary

Supplementary Methods

## Data Availability

Aggregated data that supports the findings of this study may be available upon request from the corresponding author [H.H.C.]. Individual data supporting the study’s findings are not publicly available due to participant privacy.

## Code availability

All statistical analyses were conducted using SAS software (Version 9.4; SAS Institute Inc. 2012). The code will be shared upon reasonable request by the first author [T.Y.L.].
