# Supplementary material for: Assessing overdiagnosis of fecal immunological test screening for colorectal cancer with a digital twin approach
Source: NPJ Digit Med. 2023 Feb 10;6:24. doi: 10.1038/s41746-023-00763-5 (PMC9918445; doi:10.1038/s41746-023-00763-5)
Supplement: Supplementary file 2 — Supplementary Methods [file 41746_2023_763_MOESM2_ESM.docx]

**Supplementary Methods: Estimating the proportion of over-diagnosis based on the digital twin approach**

1. Constructing the overdiagnosis-embedded multistate Markov model

The multistate-state Markov model and algebra are proposed to derive the estimates of transition rates pertaining to the overdiagnosis-embedded disease natural history of CRC, as shown in Figure 1(c). The transition rates ($\lambda_{1}$, $\lambda_{2}$) and the rate of competing risk of death ($\delta$) in this model are expressed with the following intensity matrix,

$\begin{matrix} \text{ 0 1 2 3} \\ \mathbf{Q}\left(t\right)=\begin{matrix} 0 \\ 1 \\ 2 \\ 3 \end{matrix}\left[\begin{matrix} -\lambda_{1}-\delta& \lambda_{1} & 0 & \delta\\ 0 & -\lambda_{2}-\delta& \lambda_{2} & \delta\\ 0 & 0 & 0 & 0 \\ 0 & 0 & 0 & 0 \end{matrix}\right] \end{matrix}$ (1)

State 0: Normal/ State 1: PCDP/ State 2: CP/ State 3: other cause of death (OCD)

The transition probability matrix, $\boldsymbol{P}(t)$, which is the function of abovementioned transition matrix and time interval (*t*), can be derived by using the backward Kolmogorov equation, subject to $\boldsymbol{P}(0)=\boldsymbol{I}$.

$\begin{matrix} \text{ 0 1 2 3} \\ P(t)=\begin{matrix} 0 \\ 1 \\ 2 \\ 3 \end{matrix}\left[\begin{matrix} P_{00}(t) & P_{01}(t) & P_{02}(t) & P_{03}(t) \\ & P_{11}(t) & P_{12}(t) & P_{13}(t) \\ & & P_{22}(t) & P_{23}(t) \\ & & & P_{33}(t) \end{matrix}\right] \end{matrix}$ (2)

The Markov algebra of these transition probabilities are described as follows.

$$P_{00}(t)=e^{-(\lambda_{1}+\delta)t}$$

$$P_{01}(t)=\frac{\lambda_{1}(e^{-\text{(}\lambda_{1}\text{+}\delta\text{)}t}-e^{-\text{(}\lambda_{2}\text{+}\delta\text{)}t})}{(\lambda_{2}-\lambda_{1})}$$

$$P_{02}(t)=\frac{\lambda_{1}\lambda_{2}}{\left(\lambda_{1}\text{+}\delta\right)\left(\lambda_{2}\text{+}\delta\right)}\text{+}\frac{\lambda_{1}\lambda_{2}e^{-\text{(}\lambda_{1}\text{+}\delta\text{)}t}}{\left(\lambda_{1}\text{+}\delta\right)\left(\lambda_{1}-\lambda_{2}\right)}-\frac{\lambda_{1}\lambda_{2}e^{-\text{(}\lambda_{2}\text{+}\delta\text{)}t}}{\left(\lambda_{2}\text{+}\delta\right)(\lambda_{1}-\lambda_{2})}$$

$$P_{11}(t)=e^{-(\lambda_{2}+\delta)t}$$

$$P_{12}(t)=\frac{\lambda_{2}\left(1-e^{-(\lambda_{2}+\delta)t}\right)}{\left(\lambda_{2}+\delta\right)}$$

In order to take into account removal of adenoma, the overdiagnosis-embedded disease natural history is built up as Figure 1(d), and the corresponding intensity matrix and Markov algebra of transition probabilities are expressed as follows in a similar manner.

$\begin{matrix} \text{ 0 1 2 3 4} \\ \mathbf{Q}\left(t\right)=\begin{matrix} 0 \\ 1 \\ 2 \\ \begin{matrix} 3 \\ 4 \end{matrix} \end{matrix}\left[\begin{matrix} -\lambda_{1}^{a}-\delta& \lambda_{1}^{a} & 0 & 0 & \delta\\ 0 & -\lambda_{1}^{b}-\delta& \lambda_{1}^{b} & 0 & \delta\\ 0 & 0 & -\lambda_{2}-\delta& \lambda_{2} & \delta\\ 0 & 0 & 0 & 0 & 0 \\ 0 & 0 & 0 & 0 & 0 \end{matrix}\right] \end{matrix}$ (3)

State 0: Normal/State 1: Adenoma/ State 2: PCDP/ State 3: CP/ State 4: other cause of death (OCD)

$\begin{matrix} \text{ 0 1 2 3 4} \\ P(t)=\begin{matrix} 0 \\ 1 \\ 2 \\ \begin{matrix} 3 \\ 4 \end{matrix} \end{matrix}\left[\begin{matrix} P_{00}(t) & P_{01}(t) & P_{02}(t) & P_{03}(t) & P_{04}(t) \\ & P_{11}(t) & P_{12}(t) & P_{13}(t) & P_{14}(t) \\ & & P_{22}(t) & P_{23}(t) & P_{24}(t) \\ & & & P_{33}(t) & P_{34}(t) \\ & & & & P_{44}(t) \end{matrix}\right] \end{matrix}$ (4)

where

$$P_{00}(t)=e^{-(\lambda_{1}^{a}+\delta)t}$$

$$P_{01}(t)=\frac{\lambda_{1}^{a}(e^{-\text{(}\lambda_{1}^{a}\text{+}\delta\text{)}t}-e^{-\text{(}\lambda_{1}^{b}\text{+}\delta\text{)}t})}{(\lambda_{1}^{b}-\lambda_{1}^{a})}$$

$$P_{02}(t)=\frac{\lambda_{1}^{a}\lambda_{1}^{b}e^{-\text{(}\lambda_{1}^{a}\text{+}\delta\text{)}t}}{\left(\lambda_{1}^{a}-\lambda_{1}^{b}\right)\left(\lambda_{1}^{a}-\lambda_{2}\right)}\text{+}\frac{\lambda_{1}^{a}\lambda_{1}^{b}e^{-\text{(}\lambda_{1}^{b}\text{+}\delta\text{)}t}}{\left(\lambda_{1}^{a}-\lambda_{1}^{b}\right)\left(\lambda_{1}^{b}-\lambda_{2}\right)}-\frac{\lambda_{1}^{a}\lambda_{1}^{b}e^{-\text{(}\lambda_{2}\text{+}\delta\text{)}t}}{\left(\lambda_{1}^{a}-\lambda_{2}\right)(\lambda_{1}^{b}-\lambda_{2})}$$

$$\begin{matrix} P_{03}(t)=\frac{\lambda_{1}^{a}\lambda_{1}^{b}\lambda_{2}}{\left(\lambda_{1}^{a}+\delta\right)\left(\lambda_{1}^{b}+\delta\right)\left(\lambda_{2}+\delta\right)}-\frac{\lambda_{1}^{a}\lambda_{1}^{b}\lambda_{2}e^{-(\lambda_{1}^{a}+\delta)t}}{\left(\lambda_{1}^{a}+\delta\right)\left(\lambda_{1}^{a}-\lambda_{1}^{b}\right)\left(\lambda_{1}^{a}-\lambda_{2}\right)}+\frac{\lambda_{1}^{a}\lambda_{1}^{b}\lambda_{2}e^{-(\lambda_{1}^{b}+\delta)t}}{\left(\lambda_{1}^{b}+\delta\right)\left(\lambda_{1}^{a}-\lambda_{1}^{b}\right)\left(\lambda_{1}^{b}-\lambda_{2}\right)} \\ \text{ }-\frac{\lambda_{1}^{a}\lambda_{1}^{b}\lambda_{2}e^{-(\lambda_{2}+\delta)t}}{\left(\lambda_{2}+\delta\right)\left(\lambda_{1}^{a}-\lambda_{2}\right)\left(\lambda_{1}^{b}-\lambda_{2}\right)} \end{matrix}$$

$$P_{11}(t)=e^{-(\lambda_{1}^{b}+\delta)t}$$

$$P_{12}(t)=\frac{\lambda_{1}^{b}(e^{-(\lambda_{2}+\delta)t}-e^{-(\lambda_{1}^{b}+\delta)t})}{\left(\lambda_{1}^{b}-\lambda_{2}\right)}$$

$$P_{13}(t)=\frac{\lambda_{1}^{b}\lambda_{2}}{\left(\lambda_{1}^{b}+\delta\right)\left(\lambda_{2}+\delta\right)}+\frac{\lambda_{1}^{b}\lambda_{2}e^{-(\lambda_{1}^{b}+\delta)t}}{\left(\lambda_{1}^{b}+\delta\right)\left(\lambda_{1}^{b}-\lambda_{2}\right)}-\frac{\lambda_{1}^{b}\lambda_{2}e^{-(\lambda_{2}+\delta)t}}{\left(\lambda_{2}+\delta\right)\left(\lambda_{1}^{b}-\lambda_{2}\right)}$$

$$P_{22}(t)=e^{-(\lambda_{2}+\delta)t}$$

$$P_{23}(t)=\frac{\lambda_{2}(1-e^{-(\lambda_{2}+\delta)t})}{\left(\lambda_{2}+\delta\right)}$$

1. Learning transition parameters from data on detection modes with Markov algorithm calibrated with sensitivity
   1. Prevalent screen

$The probability of being normal=\frac{P_{00}(age)+P_{01}(age)\times(1-sensitivity)}{P_{00}(age)+P_{01}(age)}$ (5)

$Prevalent screen-detected CRC=\frac{P_{01}(age)\times(sensitivity)}{P_{00}(age)+ P_{01}(age)}$ (6)

where age represents age at the first screen.

Note that normal subjects include true negative (TN) and false negative (FN).

$TN=\frac{P_{00}(age)}{P_{00}(age)+P_{01}(age)\times(1-sensitivity)}$ (7)

$FN=\frac{P_{01}(age)\times(1-sensitivity)}{P_{00}(age)+P_{01}(age)\times(1-sensitivity)}$ (8)

where age represents age at the first screen

- 1. Subsequent screen

$Normal=TN \times\left[ P_{00}\left( t \right)+(P_{01}\left( t \right)\times\left( 1-sensitivity \right)) \right]$ (9)

$Subsequent screen-detected CRC=$

$TN \times P_{01}\left( t \right)\times\left( sensitivity \right)+FN\times P_{11}\left( t \right)$ (10)

where *t* denotes inter-screening interval.

- 1. Interval cancer

$\left[ TN \times P_{02}\left( t \right) \right]+ \left[ FN \times P_{12}\left( t \right) \right]$ (11)

where *t* denotes inter-screening interval

- 1. Refuser

$Normal=\frac{{[P}_{00}(age)\times(P_{00}\left( t \right)+P_{01}\left( t \right))+P_{01}\left( age \right){\times P}_{11}\left( t \right)]}{P_{00}(age)+ P_{01}(age)}$ (12)

$Clinical-detected CRC=\frac{[P_{00}\left( age \right)\times P_{02}\left( t \right)+P_{01}\left( age \right)\times P_{12}\left( t \right)]}{P_{00}\left( age \right)+ P_{01}\left( age \right)}$ (13)

where *t* denotes the follow-up time

Markov algorithms based on the multi-state natural history model considering with the removal of adenoma were derived in a similar manner.

1. Expected cases virtually predicted by the digital screened twin compared with observed cases from the control group

The expected CRCs predicted by the digital screened twin as indicated in the method after learning the estimates of annual pre-clinical incidence rate and annual progression rate were virtually predicted by the Markov algebra encoded with transition probabilities mirroring the identical control group with population size (N) in the absence of screening, the age of entry to study (m) and, the follow-up time year (t) with the following algorithm without considering the removal of adenoma

$S\left( t \right)=N\times\frac{P_{00}\left( m \right)\times P_{02}\left( t \right)+P_{01}(m)\times P_{12}\left( t \right)}{1-P_{02}\left( m \right)}$ (14)

P_00_(m): the probability of surfacing to free of CRC before age(m)

P_01_(m): the probability of surfacing to pre-clinical detected phase before age(m)

P_02_(m): the probability of surfacing to clinical phase before age(m)

P_02_(t): the probability of surfacing to clinical phase before time(t) since last negative screen

P_12_(t): the probability of pre-clinical detected phase to clinical phase before time(t) since last negative screen

When the removal of adenoma is considered, the corresponding expected CRCs virtually predicted with the multi-state Markov model with considering the removal of adenoma is expressed as follows.

$S\left( t \right)=N\times\frac{P_{00}\left( m \right)\times P_{03}\left( t \right)+P_{01}(m)\times P_{13}\left( t \right)+P_{02}(m)\times P_{23}\left( t \right)}{1-P_{03}\left( m \right)}$ (15)

P_00_(m): the probability of surfacing to free of CRC before age(m)

P_01_(m): the probability of surfacing to adenoma before age(m)

P_02_(m): the probability of surfacing to pre-clinical detected phase before age(m)

P_03_(m): the probability of surfacing to clinical phase before age(m)

P_03_(t): the probability of surfacing to clinical phase before time(t) since last negative screen

P_13_(t): the probability of adenoma to clinical phase before time(t) since last negative screen

P_23_(t): the probability of pre-clinical detected phase to clinical phase before time(t) since last negative screen

1. The proportion of over-diagnosed CRC

The formula for estimating the proportion of overdiagnosis is

$\left( \frac{S\left( t \right)}{U\left( t \right)}-1 \right)\times100\%$ (16)

S(t): the expected number of CRC from the digital screened twin by follow-up time t

U(t): the observed number of CRC from the control group by the follow-up time (t) projected from Taiwanese national CRC incidence rate aged 50-69 between 1998 and 2003 before nationwide service screening

1. Calculate the 95% CI of the proportion of overdiagnosis

We used the Bayesian Monte Carlo Markov Chain (MCMC) to estimate the point estimate and 95% credible interval of the incidence, progression rate and sensitivity with the non-informative prior distribution. The 95% CI of overdiagnosis was reported as 2.5 and 97.5 percentage of 100,000 simulations of parameters based on the mean and covariance of estimates from Bayesian MCMC.

1. Estimating the proportion of over-diagnosed cancers validated by two randomized controlled trials and exemplified by the UK trial on gFOBT

To validate whether the proposed Markov algorithm for leaning the parameters relevant to the overdiagnosis-embedded disease natural history is adequate for modelling stool-based test, we applied it to the empirical data derived from two RCTs on CRC screening with Hemoccult-II fecal occult blood test (gFOBT) in UK and Denmark. The merit of doing validation is that the control group based on RCT design is more reliable than the comparator derived from a service screening program. The details of study design and the results have been described in full elsewhere^1,2^. In brief, the UK screening trial recruited 61,933 subjects aged 45-74 in Nottingham from February, 1981 to 1985 and stopped screening until February, 1995 with 8.5 years of the mean follow-up time. The Denmark trial recruited 152,820 subjects aged 45-74 from August, 1985 to August, 1991 with 10 years of the mean follow-up time. Table 3 shows the detailed aggregated data by detection modes from two published articles.

Illustrated by the UK trial for the calculation of the proportion of overdiagnosis, there were 74,998 subjects in the control group. By using the estimated incidence and progression rate as shown in Table 1 and the unscreened number of CRC (U(t)) is 856, the predicted number of CRC during 8.5 years of follow-up using the equation (14) can be calculated as follows:

$$S\left( t \right)=N\times\frac{P_{00}\left( m \right)\times P_{02}\left( t \right)+P_{01}(m)\times P_{12}\left( t \right)}{1-P_{02}\left( m \right)}$$

$$=74,988\times\frac{P_{00}\left( 61 \right)\times P_{02}\left( 8.5 \right)+P_{01}(61)\times P_{12}\left( 8.5 \right)}{1-P_{02}\left( 61 \right)}$$

$=74,998\times\frac{0.9142\times0.0084+0.0039\times0.9479}{1-0.0819}$

$=$931

The proportion of overdiagnosis was imputed in the following expressions

(S(t)/U(t)-1) × 100%= ((931/856)-1) ×100% = 8.79%.
